# The Global Burden of Pulmonary Diseases: Most Prevalent Problems and Opportunities for Improvement

**DOI:** 10.5334/aogh.2411

**Published:** 2019-01-22

**Authors:** Juan Wisnivesky, Juan P. de-Torres

**Affiliations:** 1 The Mount Sinai Hospital, US; 2 Clínica Universidad de Navarra, Pamplona, ES

## Abstract

Diseases of the respiratory system are a leading cause of morbidity, mortality and disability worldwide. The lungs are constantly exposed to a myriad of noxious agents present in ambient air, such as particles, chemicals and infectious organisms. At least 2 billion people are exposed globally to the toxic smoke produced by combustion of biomass fuel, inefficiently burned in poorly ventilated indoor stoves or fireplaces used for cooking or warming. One billion people inhale polluted outdoor air, and another billion are exposed primarily or secondarily to tobacco smoke. As a consequence, respiratory disease is a major cause of morbidity, disability and death worldwide primarily affecting individuals of low socioeconomic status, who are exposed to crowding, environmental exposures and poor living conditions.

Globally, 4 million people die prematurely from chronic respiratory disease. The Forum of the International Respiratory Society identified five major lung problems, the “big five,” which include chronic obstructive pulmonary disease (COPD), asthma, acute lower respiratory tract infections, tuberculosis and lung cancer. These conditions are responsible for most of the global burden of lung disease. The present issue of *Annals of Global Health* provides comprehensive information about the prevalence, pathophysiology, risk factors and impact of these conditions and suggests possible public health and policy interventions to improve patients’ outcomes.

Multidisciplinary experts from multiple areas of the globe offer their perspectives on the burden of smoking and the negative impact of tobacco on lung health as well as the role of anti-smoking legislation and anti-tobacco campaigns. Two of the principal investigators of the Proyecto Latino-Americano de Investigación en Obstrucción Pulmonar (PLATINO) study, the largest population-based survey conducted in Latin America, review the prevalence of COPD, screening strategies, risk factors, the role of hypoxemia, treatment strategies, outcomes and costs of this condition in developing countries. On the same topic, an original study reports on the economic impact of severe COPD exacerbations in a high burden region in North India. A different group of researchers from India explore the relationship between quality of life and respiratory health status among iron workers. The issue also includes a comprehensive review of the international status of interstitial lung disease with a special emphasis in its relationship with air pollution and occupational exposures. Two articles review the epidemiology and management of asthma in pediatric and adult populations, a major pulmonary condition across the globe. Tuberculosis is another highly prevalent infectious lung disease that is reviewed in this issue. Finally, a paper on the global epidemiology of lung cancer is included with special attention to risk factors such as environmental exposures, indexes of lung cancer risk and the impact of early diagnosis via screening. We hope this selected collection of papers provides a comprehensive review of the global burden of lung pathology and highlights potential strategies for improving the outcomes of patients with respiratory diseases worldwide.

